# A Protest against M. Pasteur

**Published:** 1889-03-23

**Authors:** James Greenwood

**Affiliations:** 180, Brompton Road, S. W.


### EDITOR'S LETTER-BOX.

[Our Correspondents are reminded that prolixity is a great bar to publication, and that brevity of style and conciseness of statement greatly facilitate early insertion.]

### A PROTEST AGAINST M. PASTEUR.

SIR,—Will you allow me to say a few words in reference to the suggested British contribution to the Pasteur Institute? I, for one, and as the representative of a numerous following, strongly protest against any such waste of public money. I quite agree with Mr. Goschen, in his reply to Sir Henry Roscoe's request for a Government grant, when he says that the people benefited should themselves contribute. This is very proper, of course, but that the funds of the country in these days of poverty and over-taxation should be squandered on something, the perfect efficacy of which is by no means established, I must take exception to. Sir Henry Roscoe, however, has pointed out that an inspection of the list of English subjects who have been under M. Pasteur's care during the last two years makes it appear that nearly every one of these persons were in very poor circumstances, and to expect them to contribute to the funds of the Institute is impossible. That being so, then let us not forget that all the expenses of these people were defrayed for them by private or public subscription, and why should not these enthusiasts similarly provide a reward for M. Pasteur? It is very unfair that taxpayers should see their hard-earned money squandered upon such fads. Many persons (I could enumerate a score or more of eminent medical men, amongst others) have natural and conscientious scruples against such institutions. Others, moreover, utterly discredit M. Pasteur's so-called cure for hydrophobia. That the disease itself is on the decrease is apparent, that the number of deaths due to M. Pasteur's own self-invented and alternative disease, viz., "experimental rabies," are not, is, alas, only too plain. In witness of this I may state that a short time ago a list of 148 cases in which patients have died of M. Pasteur's disease was published in his own journal, the *Annals de l'Institute Pasteur*; so failure is now admitted even by the master himself. Since that publication four other deaths have occurred, one of which (a child's) was reported as late as December 26th last year in the *Medical Press.* The dreadful case of Alphonse Smardet (26), who was bitten by a dog April 26th, 1886, submitted to M. Pasteur's care a week later, and operated upon, is to my thinking the most fatal evidence of the value of Pasteurean inoculations. This young man died of hydrophobia July 28th, 1888; that is, twenty-six months after M. Pasteur's successful (?) treatment; and from the sad circumstances of this case but one inference is obvious, namely, after years of fancied security in a fool's paradise, victims of M. Pasteur's treatment may succumb to the disease against all effects of which they have been lured into the belief that they were entirely free. Trusting you will allow the importance of this matter to excuse me for trespassing upon your valuable space, I am, etc.,

JAMES GREENWOOD.

180, Brompton Road, S.W.

